# Out of date: genetics, history and the British novel of the 1990s

**DOI:** 10.1136/medhum-2020-012022

**Published:** 2021-05-10

**Authors:** Natalie Riley

**Affiliations:** English Studies, Durham University, Durham, UK

**Keywords:** English literature, Genetics, literary studies

## Abstract

This article examines the representation of human genomics in the British historical novel of the 1990s. A form which meditates on the past and its relationship to the present, the historical novel readily lends itself to the exploration of genealogy, heredity and inheritance. Forwarding an understanding of human history, and particularly of family history, as a direct and causal function of the genes, the neo-Darwinian explanation of the genome popular in the 1990s similarly advanced its own teleological relationship between past and present. Reading Jenny Diski’s *Monkey’s Uncle* (1994), A S Byatt’s *Babel Tower* (1996) and Zadie Smith’s *White Teeth* (2000), this essay argues that the historical novel provides a unique form with which to critique the deterministic view of heredity promoted by neo-Darwinism. Focusing on moments of textual anachronism, asynchronicity and repetition in these family sagas, it shows how—at its most transgressive—the historical novel imagines temporal disruptions that bring the present into contact with the past in ways that defamiliarise conventions of linearity, order and progress. Refusing the idea of human history as a single, legible line that underpins neo-Darwinian ideas of genetic inheritance, Diski, Byatt and Smith’s novels are able to interrogate both the temporal logics and cultural capital of 1990s genetic science. While the decade was shaped and defined by popular science speculation and large-scale genetic research projects, such as the Human Genome Project (1990–2003), the novels addressed in this essay ultimately suggest the lively and seductive genocentrism of the 1990s to be inadequate to the task of explaining the complexity and meaning of the lived genome. As Diski, Byatt and Smith’s novels anticipate, the question of the uses, meanings and value of the human genome sequence continue to be of relevance within our current, postgenomic era.

## Introduction

A form which meditates on the past and its relationship to the present, the historical novel readily lends itself to the exploration of genealogy, heredity and inheritance. Its exploration of the interconnection between past and present can both consolidate and disrupt existing narratives. At its most conservative, the hermeneutic gaze of the historical novel comprises a form of genealogical or teleological thinking, which allows us to imagine the past as a direct and causal explanation of the present.1 Conversely, as Elizabeth Freeman observes, the historical novel can disrupt and even refuse to connect the past legibly to the present by use of such forms as asynchrony, anachronism, anastrophe, belatedness, delay, ellipsis, flashback, surprise, compression, pause, prolepsis, repetition and reversal.2 At its most transgressive, then, the historical novel might comprise a form of temporal disruption, bringing the present into intimate contact with the past, but in a manner that defamiliarises existing understandings of linearity, progress and order.

In this essay, the disruptive potential of the historical novel is seen to offer a critique to the neat, teleological view of heredity promoted by neo-Darwinist science in the 1990s. Emphasising an understanding of human history, and particularly of family history, as ordered, teleological and sequential, the neo-Darwinian idea of the gene as singular and stable promoted a direct and causal relationship between past and present. As the neo-Darwinian ‘unit of heredity’, the gene comprised a symbol of continuity that was treated as functionally indivisible: ‘a gene travels intact from grandparent to grandchild, passing straight through the intermediate generation without being merged with other genes’.3 For Richard Dawkins, one of the founding proponents of neo-Darwinism, genes are as good as immortal, ‘leaping from body to body down the generations’, comprising a symbol of stability and continuity that was seen as representative of linear human progress, through millions of years.4 As Patricia Waugh and others have argued, in promoting a reductive and deterministic idea of genetics, neo-Darwinist ideas moved away from Darwinism, with its focus on the interdependence of embodied interactions of living organisms within their variable and shifting environments.5 Though the present, postgenomic age might arguably have recovered this awareness of the interdependence of organism and environment, the 1990s were shaped by the determinist paradigms of neo-Darwinian genomics. The popularity of a genomic approach that reduced all aspects of human life to the genes can be seen reflected in the beginning of the Human Genome Project in 1990, which aimed to unravel the secrets of human nature through a detailed examination of human genes and their functions.6 For proponents of this genomic model, then, no aspect of human existence was beyond the reach of a wholly genetic account, with genes being directly invoked to explain complex aspects of the human condition such as sexual orientation, mental illness and intelligence.7

However, if the ideological and intellectual history of science of the 1990s was thoroughly neo-Darwinian, literature, and in particular historical fiction, was not so easily seduced. As the critic Sally Shuttleworth notes in her highly influential ‘Natural History: the Retro-Victorian Novel’ (1998), the 1990s saw an increasing engagement between the historical novel and the life sciences. Paying particular attention to Graham Swift’s *Ever After* (1992) and A S Byatt’s *Morpho Eugenia* (1992), Shuttleworth’s essay considers how historical fiction of the era revisited and reconsidered the cultural impact of evolutionary theory. Reimagining the religious, social and technological upheavals of the nineteenth century, novels such as *Ever After* and *Morpho Eugenia* form part of a large corpus of neo-Victorian novels that likewise engage with the Victorian naturalists, including Penelope Lively’s *City of the Mind* (1991), Simon Mawers’ *Mendel’s Dwarf* (1997), Andrea Barrett’s *The Voyage of the Narwhal* (1998) and A S Byatt’s *The Biographer’s Tale* (2000).8

Beyond this neo-Victorian corpus, there remains a wealth of underexamined 1990s British historical novels which address the contemporary significance of scientific theories of heredity and inheritance. Works such as Penelope Fitzgerald’s *The Gate of Angels* (1990), Ian McEwan’s *Black Dogs* (1992), Hilary Mantel’s *A Change of Climate* (1994) and Margaret Drabbles’ *Peppered Moth* (2000) all engage with the long legacy of Darwin in the 20th century. Though the following analysis builds on the pioneering critical work discussed previously, it is on this body of contemporary historical fiction that this essay focuses. Exploring Jenny Diski’s *Monkey’s Uncle* (1994), A S Byatt’s *Babel Tower* (1996) and Zadie Smith’s *White Teeth* (2000), the essay examines how these novels critique and contest the epistemological authority of neo-Darwinian genetics. Like the Victorian naturalists of *Ever After* and *Morpho Eugenia*, each of these novels features a contemporary scientific protagonist who represents the cultural authority of science and, in particular, genetics. However, while their protagonists endorse the popular genetic determinism of the 1990s, the texts ultimately suggest such genocentrism to be inadequate to the task of explaining the complexity and meaning of the lived genome. As I argue, these historical novels subvert the linear teleology of neo-Darwinian genetics. Focusing on moments of textual anachronism, asynchronicity and repetition, this essay explores Diski, Byatt and Smith’s refusal of the cultural capital and temporal logics of neo-Darwinism.

## Language of life: neo-Darwinism and anachronism in *Babel Tower*

Set during the 1960s, *Babel Tower* focuses on the postwar advances in genetics, molecular biology and the computer sciences that led to the development of the interdisciplinary research programme for understanding the human mind, termed the cognitive sciences.9 For one of the novel’s principal focalisers, Gerard Wijnnobel, vice chancellor of the fictitious University of North Yorkshire, the materialist excitement that accompanied the birth of the cognitive sciences transforms the course of his professional and intellectual life. Byatt portrays Wijnnobel as part of the first wave of linguistic nativism that emerged in the late 1950s and early 1960s, and which argued that the rules of language were hard-wired within the human brain—a stance commonly understood to have originated with Noam Chomsky’s *Syntactic Structures* (1957). Chomsky proposed that complex linguistic structures are innate to the mind, comprising a universal grammar that allows for the possibility of language. As Byatt describes in *Babel Tower*:

Gerard Wijnnobel is convinced intellectually that Chomsky is right: that the human brain is born with a capacity to generate and transform language – that this is innate, not absorbed into some empty bucket or inscribed on some tabula rasa, but there in the folds of the cortex, the dendrites and synapses and axons of the neurones in the brain. The theories, both of learning and of language, that preceded this one are more interested in the way the mind is formed and shaped by society, or by learning, or by random events.10

Crucially, however, the theories that Byatt attributes to Wijnnobel concerning the nature and acquisition of language in *Babel Tower* go far beyond what Chomsky himself was willing to affirm. As the narrator observes, Wijnnobel is committed to a purely material model of the origin of language that fuses classical genetics with the sciences of mind in a manner that more broadly parallels the genomic arguments advanced by neo-Darwinian thinkers in the early 1990s.

The rise of neo-Darwinism resulted in a second wave of linguistic nativism in the last decade of the 20th century, which centred genetics as the vehicle for a universal grammar. Of this later wave of nativist thought, it is the work of Steven Pinker that comprises the most visible and influential conception of language as resulting from evolution. While Chomsky’s own ambivalence regarding Darwinism has been noted,11 the second wave did not share this scepticism and was eager to ascribe a genetic basis to human language. Neo-Darwinian thinkers such as Pinker claimed that if the grammatical structures of language were part of human biological endowment, then it would follow that such structures were acquired through a gradual process of genetically encoded evolution. Wijnnobel’s musings in *Babel Tower* thus directly echo Pinker’s contention that, ‘if there is a language instinct, it has to be embodied somewhere in the brain, and those brain circuits must have been prepared for their role by the genes that built them’.12 Like Pinker, Wijnnobel is convinced that, as a biological inheritance, language must be determined by genetics. He contends that just as ‘beavers are born knowing how to make dams, and as spiders are born with the ability to make webs, so human beings are born with the ability to speak and think in grammatical forms’.13 This analogy is a clear allusion to Daniel Dennett’s *Darwin’s Dangerous Idea* (1995), wherein the philosopher offers a similar summary of genetic inheritance: ‘in the same way that spiders make webs and beavers make dams, we make (among many other things) books’.14 Wijnnobel’s pronouncements on language in *Babel Tower* thus closely parallel the genetic sensibilities of the second wave of linguistic nativism, far more so than they do the earlier work of thinkers such as Chomsky.

Byatt’s characterisation of Wijnnobel succeeds not only in pointing to the emergence of a first wave of linguistic nativism but also its neo-Darwinian reformulation in the 1990s. This delicate balance of the historical and the contemporaneous has posed a considerable challenge for critics of Byatt’s work. In a 1996 review of *Babel Tower*, J M Coetzee remarked that much of the genetic and linguistic science in the novel was ‘out of date’ at the time of its publication, questioning the relevance of her ‘devoting so many pages to it’.15 For Alistair Brown, it is not the cultural obsolescence of Byatt’s scientific intertexts, but rather her anachronistic blending of past and present which provokes. Brown contends that Byatt’s anachronism highlights the contingent, constructed nature of science, destabilising its epistemic certainty and authority.16 To add to these previous interventions, I suggest that it is not only the anachronistic portrayal of 1990s science in a 1960s context that is of particular significance. Indeed, by alluding so closely to contemporaneous theorists such as Pinker and Dennett, Byatt’s portrayal of Wijnnobel is able to highlight how the neo-Darwinian moment of the novel’s publication is shaped by the cultural afterlife of genetic determinism. Through the chronological inconsistencies of anachronism, Byatt is able to inscribe the history of both 1960s and 1990s science in *Babel Tower*.

The motivations that underpin Wijnnobel’s own materialist convictions are founded on a highly personal and partial longing. His grandfather, Kees, was a Jewish religious scholar who devoted himself to the search for ‘the traces of the Ur-language’, the universal speech that supposedly existed in the days before Babel.17 Believing his grandfather to have thrown away his life chasing this ‘part-mystical, part-historical, part-exegetical’ project, Wijnnobel fears that ‘there was a trap, a quirk, a temptation in the nature of language itself that led people, that induced them to spend the whole of their lives on nonsense’.18 The impact that his grandfather’s quest has on the young vice chancellor is shown to be both devastating and formative. It is only the emergence of molecular genetics that convinces Wijnnobel that a truly scientific explanation of human language might be possible, provided that the intangible, divine word of creation is substituted for the materially encoded information of DNA. This transposition allows the human genome to function as the graven text of a universal, genetic book of life, that can be read and interpreted by the scientist.

Wijnnobel exemplifies how religious narratives linger in the form of metaphors, allusions and analogies. Even Pinker’s *The Language Instinct* uses the biblical story of Babel—where ‘humanity, speaking a single language, came so close to reaching heaven that God himself felt threatened’—as an analogy for his theory of a shared, biological basis to language.19 Allusions to Babel serve a similar function in Byatt’s own text; referencing both the bombastic, nativist excitement of the 1960s and 1990s, and Wijnnobel’s own hope for a hard-wired, biological basis for language. As Wijnnobel’s good friend Vincent Hodgkiss observes, the vice chancellor functions as a modern-day ‘[a]rchitect of Babel’ who is ‘intent not on chaos, but on the discovery and communication of extraordinary order’.20 The means by which Wijnnobel attempts to found and convey this ordered vision is through the neo-Darwinian synthesis of genetics and evolutionary science. Yet, in alluding so closely to the narrative of Babel, Byatt’s text implicitly undermines both Wijnnobel’s borrowed scientific authority and the deterministic certainty he envisions in the genome.

Part of the larger nature-nurture debate, the controversy surrounding the language instinct portrayed in *Babel Tower* further challenges Wijnnobel’s reliability as a source of moral authority. As Byatt highlights, the conviction in a universal, genetic basis to language provokes moral revulsion in many of Wijnnobel’s interlocuters:

To believe that linguistic competence is both innate and unalterable, in the present world, smacks of determinism, smacks of predestinarianism, and of more unpleasant things, a suggestion that heredity not environment, differentiates between men. Many of the men Wijnnobel meets find that suggestion morally repugnant, exactly as he found his father’s ideas repugnant.21

By alluding to the close relationship between genetic determinism and the spectre of eugenics, *Babel Tower* gestures to the sociopolitical concerns that accompany the genocentric scheme of human behaviour. While Wijnnobel is portrayed as disturbingly indifferent to the potential sociopolitical consequences of the science he promotes, the characters around Wijnnobel are able to express the wider, cultural meaning of his neo-Darwinian ideas. As Richard Lewontin and others have noted, in positing genetics as the basis of human society, genetic determinism all too often naturalises social inequalities, rendering them as the result of biology and thus, in practical terms, unalterable.22 Satirising its principle advocate for genetic determinism, *Babel Tower* shows Wijnnobel to be an ultimately partisan and flawed figure. This tension between narrative viewpoints in Byatt’s text thereby expresses a genomic scepticism that applies not only to the blind faith in genetic possibilities present in the heyday of classical genetics but also to the genomic moment of the 1990s when the novel was published.

## Eugenic afterlives: repetition and racialised science in *White Teeth*

Zadie Smith’s *White Teeth* likewise addresses the lingering legacy of eugenics within contemporary genetics, exploring the latter’s potential to engender new forms of discrimination. Set primarily in the latter half of the 20th century, yet moving freely between 1857 and 1999, Smith’s saga narrates the lives and loves of three families, the Iqbals, the Joneses and the Chalfens. *White Teeth* thus moves beyond *Babel Tower*’s focus on the politics of genetics within the laboratory and the university, to also explore its impact in the wider milieu of the household and the playground. In so doing, Smith illustrates how the laboratory and the university are unavoidably entangled within the wider communities in which such institutes are embedded. The fates of all three families, then, are shown to be enmeshed with the novel’s exploration of race and racialised science, just as that very science is portrayed as being inextricably in relation with the various families that comprise the wider community of Smith’s novel.

The unavoidable nature of such entanglement is particularly notable in the final scene of *White Teeth*, when the Iqbals, the Joneses and the Chalfens all gather together at a press event, held on 31 December 1992, for Marcus Chalfen’s genetic project, ‘FutureMouse’. The principal figure of the scientist in Smith’s novel, Chalfen works at the fictional Perret Institute, where he has spent decades experimenting in the field of genetic engineering. FutureMouse is the culmination of his work—a designer mouse which is genetically programmed to die of cancer on a known date, New Year’s Eve, 1999. The aim of its creation is to prove that randomness and mutation can be eliminated from the genome via bioengineering. Though the gathering portends to celebrate this scientific breakthrough and the future possibilities Chalfen believes it to afford, it is instead the determining weight of history repeating itself that shapes the novel’s climax. Past ruptures into present, and the racialised science of eugenics re-emerges in the supposedly hygienic and apolitical form of contemporary bioengineering, undermining the cultural authority of Chalfen’s science and mocking the erasure of contingency that his press conference is intended to announce.

Despite Chalfen’s repeated assurances over the course of the narrative regarding the apolitical nature of his work, it becomes apparent that his science possesses an inescapably political dimension whose meaning he cannot control or dismiss. The institute at which he worked was founded by an alleged Nazi collaborator, Dr Pierre Perret. Captured by Archie Jones and Samad Iqbal during World War II, Perret is led into the woods for a summary execution. After escaping from his captors, the reader learns that he emigrated to London, where he proceeded to found the Perret Institute for advanced genetic study. It is Chalfen himself who stresses the intellectual debt that he feels he owes to Dr Perret, characterising the latter as ‘a personal inspiration’ and ‘mentor’ who ‘laid the foundations for so much of this work’.23 Yet, it is precisely this acknowledgement and the re-emergence of Perret that reveals the extent to which the present of Smith’s novel, and its cutting-edge genetic science, is still haunted by a eugenicist past. As Jerome De Groot observes, historical fiction is a form which gives ‘voice’ to ‘ghosts within the now, echoes and revenants of history, which still linger and exert a modern-day shaping influence.24 Perret, or Dr Sick as he is referred to in the closing days of World War II, thus functions as the revenant of *White Teeth*, literally and metaphorically brought back from the dead, whose influence can still be felt in the institute he founded, and the genetic work it champions.

Obliviousness on Chalfen’s part to questions of racialised science is a recurring theme in Smith’s novel. As Josie Gill observes, Chalfen is generally blind to the presence of racism, such as when, for example, he fails to realise the charged, racial microaggressions present within his own household.25 For Gill, ‘the scientific removal of the significance of race from genetics comes from a position of (often white) privilege which does not recognise the ways in which racism is deeply ingrained in all parts of society’.26 Thus, Marcus Chalfen and his wife, Joyce, espouse a series of racial microaggressions during their interactions with Irie Jones and Millat Iqbal, treating them, as Gill notes, with a blend of paternalism and sexual exoticism.27 Marcus Chalfen pantomimes the ‘foreign syllables’ of ‘Mill-yat Ick-Ball’,28 while Joyce remarks that he is ‘gorgeous’ and ‘Like Omar Sharif thirty years ago’.29 Irie Jones is treated in a similar manner as a ‘big brown goddess’,30 of whom Marcus Chalfen scathingly remarks that she lacks the ‘head’ for ‘hard science’31: only conceding that ‘she’s sharp in a way, but it’s the menial work, the hard grafting, that she’s good at – she’d make a lab assistant maybe’.32 The Chalfens, then, in Smith’s novel, function as a collective caricature of the heteronormative logics of the white, middle-class family, within a reproductive futurity anchored in a linear, deterministic and racist understanding of inheritance and progress. They are, reports Irie Jones, ‘[l]ike clones of each other’, and ‘their dinner table was an exercise in mirrored perfection, Chalfenism and all its principles reflecting itself infinitely’.33 This image of uninterrupted Chalfenism, receding into eternity, is exemplified best when Joyce Chalfen parades the daguerreotypes of her ‘grand old family’ in front of Clara and Irie Jones.34 As historical artefacts, the daguerreotypes participate in the work of constructing history: providing a material means of reinforcing a teleological relationship between past and present, rooted in a linear, deterministic understanding of genetic inheritance. Standing in front of the picture of Marcus’ grandfather, Dr Solomon Chalfen, Joyce announces that the ‘first time Marcus showed me that picture, I knew I wanted to marry him’.35 Joyce characterises her husband’s appeal as precisely that of stability and linear, teleological progress, anchored in a belief in the direct, genetic inheritance of character and intellect. As she goes on to note, making this eugenic subtext explicit: ‘you’ve got to suspect it’s in the genes, haven’t you? All these brains. I mean, nurture just won’t explain it’.36 For Joyce, as for Marcus himself, their family history is indicative of their heteronormative future: a historical, reproductive sequence, whose continuation will beget further white, middle class, Chalfen ‘intellectuals’, in the unceasing transmission of the very genetic material that has supposedly fashioned such individuals.37

The Chalfens thus embody what Elizabeth Freeman characterises as chronobiopolitics, a term which describes the normative social, familial and psychological structures that support the standard, linear arrangement of time. Chronobiopolitics offers an ordered and sequential progression of events which make up the teleological schemes of living, including ‘marriage, accumulation of health and wealth for the future, reproduction, childrearing, and death’.38 Freeman’s definition of reproductive futurity, then, implicates both capitalism and heterosexuality into the logics of inheritance, yet it also highlights the role played by inheritance as the basis of a naturalised, sociopolitical order. These arrangements of living, in turn, structure the logic of inheritance: ‘rather than just the transfer of private property along heteroreproductive lines, inheritance becomes the familial and collective legacy from which a group will draw a properly political future—be it national, ethnic, or something else’.39 In the case of the Chalfens, this political future is anchored in the concept of direct genetic transmission and its determining influence on intellect, character and opportunity.

The genealogical thinking of Marcus Chalfen and his family, rooted in a narrow, deterministic conception of genetic inheritance, and supportive of the pervading chronobiopolitical regime, exists in tension with the more mutable understanding of Irie Jones. Whereas Joyce Chalfen revels in a linear, teleological understanding of inheritance and the generational privilege it would entail, for Irie Jones, the question of deterministic, genetic inheritance is more troubled. Jones is ‘intent on fighting her genes’, which she views as an unwanted reminder of her otherness.40 A lack of representation within her wider culture leads her to feel like she is a misfit: ‘There was England, a gigantic mirror, and there was Irie, without reflection. A stranger in a strange land’.41 Though, like Joyce Chalfen, Irie Jones uses the language of heredity, it is not similarly deployed to express satisfaction with a teleological conception of genetic transmission. Rather, it is used to name the enemy that she feels she must overcome—Jones is ‘intent on transformation, intent on fighting her genes’.42 She is depicted as ‘unwilling to settle for genetic fate; waiting instead for her transformation from Jamaican hourglass […] to English Rose’.43 This attempt to refuse ‘roots’ seems most obviously realised in the birth of Irie’s daughter: a child who ‘can never be mapped exactly nor spoken of with any certainty’.44 For Jones, her child, whose father could be either Majid or Millat Iqbal, seems to live beyond genetic prescription of the kind embraced by the Chalfens.45

Yet, though Irie Jones’ child hints at the possibility of a more heterogenous future, the present of the novel portrays her daughter as the lone exception to the seemingly deterministic rule of history and heredity. Rather than the child and the future she perhaps represents, it is Marcus Chalfen, racialised science and the idea of history repeating itself with which the novel concludes. Portrayed as at best oblivious, or at worst indifferent, to his mentor’s embrace of eugenics and racialised science, Marcus Chalfen is repeatedly shown to believe that science is a privileged form, whose meaning is separate from any concept of racialisation. Even when the potential for racism in science is directly pointed out to him—for example, during an encounter with an unnamed reader of his work at an airport—he still fails to recognise the racial thinking which remains operative in his own discipline: ‘You’ve got to be seriously naïve if you don’t think the West intend on using this on the East, on the arabs’.46 Calling into question Chalfen’s thinking about science in a contextless fashion, the nameless reader offers a stark warning of the dangers inherent in such developments in genetic engineering, if they are not accompanied by an acute awareness of the risks that they can pose:

They talk about leaps and bounds in the field of medicine yada yada yada, but bottom line, if someone knows how to eliminate “undesirable” qualities in people, do you think some government’s not going to do it? I mean what’s undesirable? There’s just something a little fascist in the whole deal … I guess it’s a good book but where are we going here? Millions of blonds with blue eyes? Mail order babies? I mean, if you’re Indian like me you’ve got something to worry about, yeah?47

It is only Chalfen’s privilege, both of class and ethnicity, and a self-serving commitment to the supposedly apolitical nature of science, that allows him to dismiss this encounter as merely a manifestation of ‘the usual neo-fascist tabloid fantasies’.48 Secure in his privilege and self-regard, Chalfen can ignore his reader’s worries concerning the possibility of genetically policing sexual and racialised characteristics. Much like *Babel Tower*, then, *White Teeth* underscores the naivety and limitations of its principal advocate for genetic determinism in his refusal to engage with the wider meaning of genetic science.

As with Byatt’s portrayal of Gerard Wijnnobel, Smith’s novel likewise presents Marcus Chalfen’s own understanding of genetics and its cultural influence as dangerously, even comically, irresponsible. When issues surrounding the potential political and social meaning of science are mooted, Chalfen, like Byatt’s vice chancellor, is all too quick to disregard such conjecture as mere social constructivism—the work of ‘strange French men who think truth is a function of language, or that history is interpretive and science metaphorical’.49 Chalfen’s caricature of social constructivism fails, however, to fully acknowledge or adequately engage with a critique of science as wholly objective and separate from its social context. As in *Babel Tower*, then, the inability of the scientist to perceive the cultural meaning of the genetic breakthroughs he pursues undermines the epistemological authority of science within the novel and remains an abiding source of narrative tension.

## Metaphorical Science: genetics, information and analogy

Science studies has done much to show how metaphors have influenced the history of science, shaping research questions, models and practices.50 As Lewontin observes, given the constitutive power of metaphor, scientists must be ‘conscious of the metaphorical content of our words and not be carried away when we write of the “cell machinery” which “reads” the DNA during the process of “development”’.51 As both Lily Kay and Evelyn Fox Keller have shown, the particular metaphors that were used to illustrate the relationships between DNA, RNA, proteins and organismic bodies were informatic in nature.52 For Kay, this representation of heredity, and indeed life, in terms of information, did not derive from the internal logics of genetics, nor did it comprise an outcome of the elucidation of the architecture of the double-helix in 1953.53 Rather, she contends, the linguistic tropes and textual metaphors of the life sciences that were central to the semiotic formulation of the genetic code were transported into molecular biology from cybernetics, information theory, electronic computing, and control and communication systems—technosciences that were deeply embedded within military experiences of World War II and the Cold War.54 As she notes, notions of information, message and code were being inscribed into biology and genetics as early as the mid-1940s, accompanying the rise of information theory, cybernetics and computing.55

These informatic metaphors in turn serve to reinforce conceptions of genetic determinism and reductionism, promoting a linear model of life in which DNA operates as script, blueprint and information cache. As Fox Keller notes, in a computer age, informatic metaphors meant that DNA could be imagined as a software programme executed in the creation of an organism.56 Crucially, as Fox Keller observes, such metaphors also led to an insistence on unidirectional causality, (and) its repudiation of the possibility of a substantive influence on genes, either from their external or from their intracellular or intercellular environment. Instead of circular feedback, it promised a linear structure of causal influence, from the central office of DNA to the outlying subsidiaries of the protein factory. To this end, they appropriated the cybernetic term information but used it in its colloquial rather than in its technical sense.57

The very metaphors, then, through which DNA was conceived, in turn shaped the nature of the relationship that was imagined between gene, organism and environment, more so than any evidentiary basis. Even in the present moment, when a genomic understanding of DNA is increasingly being superseded by a postgenomic conception that emphasises epigenetic factors, the lingering influence of informatic metaphors can still be observed. As Jenny Reardon and others have noted, the postgenomic era is one that is even more informed by the logics and practices of information than the periods of genomic history that preceded it.58 In an era of extreme globalisation and ‘informatic capitalism’, biotechnological enterprise has become inextricable from the operations of the wider regimes of capital.59 Biomedicine now reaches into the lives of people as it never has before, in the form of human genome databases and biobanks that facilitate the marketing of inexpensive and consumer-targeted sequencing and screening techniques.60 In this respect, the conception of DNA as a form of biological instruction has never really abated. If anything, the growing transformation of information into a commodity has only encouraged the dominance of informatic metaphors within the field of biomedicine.

In *Babel Tower*, it is Wijnnobel’s friend, Vincent Hodgkiss, who draws attention to the potential dangers and intellectual pitfalls of the use of informatic analogies in regard to genetic thinking. When Wijnnobel hosts a dinner party, he gathers together a range of scientists whose fields of expertise span the cognitive sciences, including neurochemistry, psychology and artificial intelligence. One of the principal topics of conversation among the attending academics is the possible existence of a memory molecule or ‘elusive engram’.61 Popularised by the pioneering psychologist Karl Lashley, the concept of an engram functions as a placeholder for the theoretical possibility that memory, as information, is somehow stored directly within the nervous system in some material form. It is this viewpoint which, the narrator observes, is broadly, if cautiously, endorsed by the gathered cognitive scientists:

The idea is that it is possible that learned information, as well as genetic coded information, might be retained in and transmitted by very large molecules, such as the DNA and the RNA. And this idea received reinforcement from the immunological study of proteins, since antibodies recognise intruders into organisms, remember them, encode the information in some way, and prepare themselves to resist subsequent invaders. So we wonder in turn, if the roots of our own memories, the structure of our own consciousness, are to be found in these amazing macromolecules.62

The conception of genetic material that such a possibility relies on is that of an information cache, encoded using the four-letter language of the DNA base pairs: an understanding that, in emphasising the equivalence of information above all, suggests that it is possible that complex phenomena, such as memory, or indeed language and consciousness, could be directly encoded within an organism’s genome as yet another form of biologically inscribed information.

While there emerges a broad, if tempered enthusiasm for the eventual discovery of the engram among those gathered at Wijnnobel’s dinner party, the principal dissenting voice comes not from the attending cognitive scientists, but rather from Vincent Hodgkiss. A student of the humanities, whose own area of research concerns the study of Wittgenstein, Hodgkiss is highly critical of the scientific speculation engaged in by the other guests:

The question is, whether the word information means the same in all cases, that of immunology, that of DNA, that of the mind of the scientist building a computer, or whether you are all thinking by analogy, which is dangerous.63

For Hodgkiss, the peril posed by analogy is clear. While thinking of DNA as an encoded language conceptualises a highly complex aspect of scientific inquiry, it also highlights the danger of how seductively imprecise such an analogy can be. Imprecision, for Hodgkiss, can in turn lead to the drawing of false equivalences—in this case, that information means the same thing in each instance under discussion. Byatt’s treatment, via Hodgkiss, of the informatic analogies concerning the DNA molecule thus parallels the critiques voiced within science and technology studies such as those discussed earlier. It highlights how the dangers inherent in a belief that DNA constitutes information above all else were present in the early days of classical genetics and have only become more entrenched in the intervening decades. Both *Babel Tower* and *White Teeth*, then, serve to illustrate how the particular meaning of the ‘information’ contained within DNA is highly contextual in nature and shaped in relation to a given environment and its sociopolitical concerns.

## All her life: meaning and the asynchronous in *Monkey’s Uncle*

Jenny Diski’s *Monkey’s Uncle* likewise offers a profoundly critical portrayal of the meaningfulness of considering DNA as information. A surrealist, historical novel, Diski’s text is divided into three interwoven narrative strands centred around the fictitious geneticist, Charlotte FitzRoy, and the historical figures Charles Darwin and Captain Robert FitzRoy. The novel’s first strand, set in 1990, focuses on a sustained period of mental ill health for Charlotte FitzRoy, following the sudden death of her daughter. Consumed by the delusion that she is the direct descendant of Captain Robert FitzRoy, Charlotte FitzRoy, like Gerard Wijnnobel and Marcus Chalfen, is seduced by the explanatory potential of a linear, deterministic understanding of genomic inheritance. She seeks to explain her own experiences of depression by appealing to her supposed genetic inheritance. Reflecting this neat, teleological relationship of past and present, is the inclusion of a second narrative strand which traces Robert FitzRoy’s voyages on the HMS Beagle with the young Charles Darwin in the 1830s and his subsequent suicide in 1865. Yet, complicating any neat genealogy of past and contemporaneous present is the inclusion of the novel’s asynchronous, third strand, in which Charlotte FitzRoy herself encounters an ageing Darwin. Through this use of asynchronicity, *Monkey’s Uncle* both explores the explanatory potential of a neo-Darwinian understanding of inheritance and disrupts the established temporal order it would require, blurring any linear, causal and genealogical relationship of Victorian past and late-20th century present.

It is through such linear, genomic understandings of inheritance that Charlotte FitzRoy initially attempts to explain her experiences of depression and mental ill health. She becomes obsessed by the notion that she is a recipient of Robert FitzRoy’s genetic madness:

So much of FitzRoy’s story, his character, chimed in with details of her past she was more or less convinced by the time she had finished her first reading of the book, and when she looked at the pictures of FitzRoy young and old, and also of Castlereagh, she could no longer doubt that she was a FitzRoy. The physical evidence was compelling – the grey eyes, the sharp nose, the receded chin. They were all present in her, and, she could see, to some extent in both Julian and Miranda. She couldn’t deny that they must have those chromosomes in common, but it was other, less evident chromosomes which gripped her mind.64

Charlotte FitzRoy thus attempts to justify her belief by reference to a series of observable physical traits—‘grey eyes and aquiline noses’—of whose direct genetic origin and expression she can be reasonably certain, as well as the fact that comparable genetic material must be shared by herself and her two children.65 Having established the probable genetic similarity of such physical traits, however, Charlotte FitzRoy then attempts to infer the presence of shared character traits, such as ‘a statistical tendency to cut their own throats’, as being likewise founded in a genomic understanding of genetic inheritance.66

It is in attempting to substantiate this latter assertion of a shared, genetic character between Charlotte FitzRoy and her supposed ancestor, that the explanatory power of her genomic understanding of inheritance begins to break down. Convinced that ‘the laboratory now seemed the only place where real answers to her questions might be found’, she attempts to locate the specific gene that she believes to be responsible for her familial history of mental ill health.67 FitzRoy’s hopes, however, are dashed by the sheer scale of the task before her, and she quickly becomes frustrated with the lack of meaning inherent in genetic material. As the narrator observes, ‘the messages were there, all right, and yet they didn’t speak’.68 Contemporaneous technologies of genetic sequencing allow Charlotte FitzRoy, ‘like a wanton boy’, to take her chromosomes and ‘break them apart piece by piece, stain them, slice them, clone them, and yet never find the way to put them back together that would amount to the story she was looking for’.69 The narrative which Charlotte FitzRoy wishes to construct, then, is one that relies on a genomic understanding of DNA as a form of information with an inherent historical meaning, connecting her to the past through the linear continuum of genetic inheritance:

Increasingly, it was Charlotte’s silent, self-obsessed belief that she had been doomed from the moment she was born. From before that moment, in fact. Her doom, if it was true, could be traced back (with the benefit of hindsight and an informed guess on the basis of those grey eyes and aquiline nose) to 1769 at least.70

Though Charlotte FitzRoy, like Gerard Wijnnobel, was convinced that ‘somewhere’ among her genome was ‘the truth, a pattern, an explanation, the story of Charlotte’s future and history’s past’, the task of attributing a stable meaning to this vast volume of genetic information, independent of environmental influence, proves to be beyond her capacity to decipher or inscribe.71 As she is finally forced to concede, the karyotype ‘pictures she had made of her innermost self were, in reality, useless as a practical aid, and the sense of inevitable doom went on increasing in spite of the hours she spent staring at the evidence’.72 Though Charlotte FitzRoy may wish otherwise, the genetic language that she seeks to understand has no certain meaning absent of its referent. Viewed in the abstract, she lacks the environmental context necessary to begin making sense of the sheer volume of genetic material with which she is confronted: ‘it was like looking for a meaningful message in an infinitely long tickertape produced by a monkey on a typewriter with only four keys’.73 By the conclusion of Diski’s narrative, FitzRoy is portrayed as having largely abandoned any genomic understanding of genetic inheritance.

The depth of Charlotte FitzRoy’s intellectual transformation is perhaps best illustrated in the trajectory of her asynchronous interaction with Charles Darwin, on whose work the foundation of her initial, neo-Darwinian, genomic obsession once rested. FitzRoy is portrayed as being initially enamoured by her discussions with Darwin, which encompass politics, religion and society, and reflect her intellectual indebtedness to his work. By the conclusion of the narrative, however, Charlotte FitzRoy is shown to have become discontented with the meaning and value of Darwin’s theories, and with the disdain which he shows for the beliefs and struggles of her putative ancestor. Defending Robert FitzRoy’s emotional investment in a caring creation, Charlotte FitzRoy argues that Darwin’s theory of evolution, though supported by developments in modern genetics, was still not necessarily a social right. As FitzRoy notes, the ‘poor are still poor, the confused are still confused, and science has provided no more comfort or certainty than religion did’.74 Just as with *Babel Tower* and *White Teeth*, in Diski’s novel the seductive explanatory potential of neo-Darwinism is presented, but a tension remains between the reductive teleology it offers and the wider social questions of its value and meaning. In its very asynchronicity, Charlotte FitzRoy’s encounter with Darwin comprises a temporal disruption, which interrupts the notion of science’s teleological and sequential progress, undermining its claims to authority and challenging what such transhistorical scientific paradigms can mean, absent of their social context.

## Postgenomic information and the continued problem of knowledge

These unsettled issues of the meaning of genetic information, explored in the three novels discussed, have carried over into the postgenomic age. The term ‘postgenomic’ applies broadly to any biological research after the completion of the major genome projects that employs genomic technologies and draw on genomic knowledge.75 Among the many possibilities presented by the decoding of the human genome has been the expansion of the type and availability of genetic analysis. The falling cost and increasing speed of whole-genome technologies means that these techniques are now ubiquitous in a variety of private and public enterprises.76 Such whole-genome technologies include human genome databases and biobanks; microarray chips for assessing the expression of hundreds of thousands of genes in human tissue; rapid, inexpensive, next-generation genome sequencing technologies; bioinformatic and computational advances in genome-wide association studies; and direct-to-consumer, mail-order mass sequencing and genome analysis facilities.77 Such information derived from genetic science has now moved out of the rarefied and specialised domains of biomedicine, into more general cultural circulation where it forms a key part of a biologically informed, public discourse of self-fashioning.

Tracking the broader, social transmission of the logics and techniques of genetic science—the social life of DNA—we can apprehend how genetics is increasingly relied on to answer fundamental questions, not only about human identity but also about national and political community, social justice and collective memory.78 Jenny Reardon summed up this postgenomic condition as the struggle of meaning making:

Now that we have “the human genome” sequence, what does it mean? Tremendous feats of biomolecular engineering produced the sequence. However, what was the route between this technological feat and meaningful knowledge that might foster life and human understanding? In the decade after the completion of the HGP, this turn to the question of meaning—the question of the uses, significance, and value of the human genome sequence—marks what I call the postgenomic condition.79

Indeed, the question of the meaning of genetic information has become even more important in a postgenomic age which, in the era of big data, is one increasingly divorced from the human scale. As Reardon proceeds to explain, the making of meaning seems an ever-more necessary and precarious topic, now ‘that the task is no longer sequencing the human genome but interpreting it’.80 The reliance in the biological sciences on digital computing and data management results in a ‘bias toward automated approaches—such as whole genome analyses—[that] affects what we can know about genomes and the quality with which we know it’.81 Computers and informatics might provide excellent tools for storing, managing and looking for patterns in massive amounts of genomic data, but the transformation of this genomic data into meaningful knowledge still requires humans to make judgements about which algorithms to use and about which data to input.82

In highlighting the difficult and contentious nature of the conversion of genetic data into meaning, the three novels addressed in this essay all use the historical fiction form as a means of illustrating how the question of the meaning of genetics is one of contested translation. From the playground to the university, the laboratory to the airport, the potential meanings of genetics are shown to circulate in society and are used to inform choices and understandings surrounding questions of identity, family and nationality. Through literary techniques such as asynchronicity, anachronism and repetition, the novels in this study both highlight the seductive, explanatory potential of a linear, deterministic understanding, and refuse any simple or singular teleology of genetics. In so doing, they show how the legibility of genetic data and stable inscription of meaning remain contested. Neither history nor genetics are shown to be settled, but rather function as palimpsests whose significance must be interpreted and curated. Though these texts are not in themselves postgenomic—they do not imagine the postgenomic era to come—they do highlight how the logics and cultural capital of genetics has always been, and must continue to be, disrupted, refused and undermined.

## Data Availability

Data sharing is not applicable as no datasets were generated and/or analysed for this study.

## Notes

1. See De Groot (2008), *Consuming History: Historians and Heritage in Contemporary Public Culture* (London: Routledge, 2008), Freeman (2010), *Time Binds: Queer Temporalities, Queer Histories* (Durham; NC: Duke University Press, 2010).

2. Elizabeth Freeman, *Time Binds*, xxii.

3. Dawkins (1976), *The Selfish Gene* (Oxford: Oxford University Press, 1976), 11 and 34.

4. Dawkins, *The Selfish Gene*, 34.

5. Waugh (2011), ‘Mind in Modern Fiction: Literary and Philosophical Perspectives after Darwin,’ in *The Evolution of Literature: Legacies of Darwin in European Cultures*, ed. by Nicholas Saul and Simon J. James (Amsterdam: Rodopi, 2011), 125–40.

6. Hanson (2020), *Genetics and the Literary Imagination* (Oxford: Oxford University Press, 2020), 1.

7. Sarkar (1998), *Genetics and Reductionism* (Cambridge: Cambridge University Press, 1998), 2.

8. For a further discussion of science in the neo-Victorian novel see, Glendening (2013), *Science and Religion in Neo-Victorian novels: Eye of the Ichthyosaur* (New York; London: Routledge, 2013).

9. For a detailed history of the emergence of the cognitive sciences see, for example, Gardner (1987), *The Mind’s New Science: A History of the Cognitive Revolution* (New York: Basic Books, 1987), or Rose and Abi-Rached (2013), *Neuro: The New Brain Sciences and the Management of the Mind* (Princeton; Oxford: Princeton University Press, 2013).

10. A. S Byatt (1997), *Babel Tower* (London: Vintage, 1997), 192.

11. Noam Chomsky (1972), *Language and Mind* (San Diego: Harcourt, Brace, and World, 1972), 97.

12. Pinker (1994), *The Language Instinct: The New Science of Language and Mind* (London: Allen Lane, 1994), 299.

13. Byatt, *Babel Tower*, 192.

14. Dennett (1995), *Darwin’s Dangerous Idea: Evolution and the Meanings of Life*. (London: Allen Lane, 1995), 135.

15. Coetzee (1996), “En Route to the Catastrophe,” *The New York Review of Books*, 43:10 (6 June 1996), 17-19 (18).

16. See Brown (2007), “Uniting the Two Cultures of Body and Mind in A. S. Byatt’s *A Whistling Woman*,” *Journal of Literature and Science*, 1:1 (2007), 55–72 (61). See also Alexa Alfer and Amy Edwards de Campos (2013), *A. S. Byatt: Critical Storytelling* (Manchester: Manchester University Press, 2013), 82.

17. Byatt, *Babel Tower*, 190.

18. Byatt, *Babel Tower*, 190.

19. Pinker, *The Language Instinct*, 16.

20. A. S Byatt (2003), *A Whistling Woman* (London: Vintage, 2003), 327.

21. Byatt, *Babel Tower*, 192-3.

22. Lewontin et al. (1982), “Bourgeois Ideology and the Origins of Biological Determinism,” *Race and Class*, 24:1 (1982), 1-16 (5).

23. Smith (2002), *White Teeth* (London: Penguin, 2002), 531.

24. De Groot (2016), *Remaking History: The Past in Contemporary Historical Fictions* (London: Routledge, 2016), 74.

25. Gill (2020), *Biofictions: Race, Genetics, and the Contemporary Novel* (London: Bloomsbury, 2020), 92.

26. Gill, *Biofictions*, 92.

27. Gill, *Biofictions*, 91.

28. Smith, *White Teeth*, 317.

29. Smith, *White Teeth*, 320.

30. Smith, *White Teeth*, 329.

31. Smith, *White Teeth*, 386.

32. Smith, *White Teeth*, 386.

33. Smith, *White Teeth*, 314.

34. Smith, *White Teeth*, 353.

35. Smith, *White Teeth*, 353.

36. Smith, *White Teeth*, 354.

37. Smith, *White Teeth*, 354.

38. Freeman, *Time Binds*, 4.

39. Freeman, *Time Binds*, 5.

40. Smith, *White Teeth*, 273.

41. Smith, *White Teeth*, 266.

42. Smith, *White Teeth*, 273.

43. Smith, *White Teeth*, 266-7.

44. Smith, *White Teeth*, 527.

45. Smith, *White Teeth*, 527.

46. Smith, *White Teeth*, 417.

47. Smith, *White Teeth*, 418.

48. Smith, *White Teeth*, 419.

49. Smith, *White Teeth*, 312.

50. See Dupuy (2000), *The Mechanization of the Mind: On the Origins of Cognitive Science*, trans. M. B. DeBevoise (Princeton: Princeton University Press, 2000), 78; Oyama (1985), *The Ontogeny of Information: Developmental Systems and Evolution* (Cambridge: Cambridge University Press, 1985), 46-72; and Kay (2000), *Who Wrote the Book of Life? A History of the Genetic Code* (Stanford: Stanford University Press, 2000), 22–23.

51. Lewontin (1985), “Foreword,” in Oyama, *The Ontogeny of Information*, vii–xv (xv).

52. Fox Keller (2002), *Making Sense of Life: Explaining Biological Development with Models, Metaphors, and Machines* (Cambridge: Harvard University Press, 2002), 93; Kay, *Who Wrote the Book of Life?*, 22–23.

53. Kay and Kay (1995), “Who Wrote the Book of Life? Information and the Transformation of Molecular Biology, 1945-55,” *Science in Context*, 8:4 (1995), 609–34 (611).

54. Kay, “Who Wrote the Book of Life?,” 611–2.

55. Kay, “Who Wrote the Book of Life?,” 629.

56. Fox Keller (1995), *Refiguring Life: Metaphors of Twentieth Century Biology* (New York: Columbia University Press, 1995), 93.

57. Fox Keller, *Refiguring Life*, 94–95.

58. Sunder Rajan (2006), *Biocapital: The Constitution of Postgenomic Life* (Durham: Duke University Press, 2006), 16; Reardon (2017), *The Postgenomic Condition: Ethics, Justice, and Knowledge After the Genome* (Chicago: The University of Chicago Press, 2017), 38.

59. Reardon, *The Postgenomic Condition*, 27.

60. Reardon, *The Postgenomic Condition*, 38.

61. Byatt, *Babel Tower*, 251.

62. Byatt, *Babel Tower*, 250.

63. Byatt, *Babel Tower*, 250-51.

64. Diski (1994), *Monkey’s Uncle* (London: Phoenix, 1994), 174.

65. Diski, *Monkey’s Uncle*, 34.

66. Diski, *Monkey’s Uncle*, 36.

67. Diski, *Monkey’s Uncle*, 174.

68. Diski, *Monkey’s Uncle*, 15.

69. Diski, *Monkey’s Uncle*, 15.

70. Diski, *Monkey’s Uncle*, 177.

71. Diski, *Monkey’s Uncle*, 178.

72. Diski, *Monkey’s Uncle*, 178.

73. Diski, *Monkey’s Uncle*, 176.

74. Diski, *Monkey’s Uncle*, 185.

75. Richardson (2011), “Race and IQ in the Postgenomic Age: The Microcephaly Case,” *BioSocieties*, 6:4 (2011), 420–46 (421–2).

76. Nelson (2012), “Reconciliation Projects: From Kinship to Justice,” in *Genetics and the Unsettled Past: The Collision of Race, DNA, and History*, ed. by Keith Wailoo, Alondra Nelson, and Catherine Lee (New Brunswick; NJ: Rutgers University Press, 2012), 20–31 (20).

77. Stevens and Richardson (2015), “Beyond the Genome” in *Postgenomics: Perspectives on Biology After the Genome*, ed. by Hallam Stevens and Sarah Richardson (Durham; NC: Duke University Press, 2015), 1–8 (2).

78. Nelson, “Reconciliation Projects,” 20.

79. Reardon, *The Postgenomic Condition*, 2.

80. Reardon, *The Postgenomic Condition*, 198.

81. Reardon, *The Postgenomic Condition*, 198.

82. Reardon, *The Postgenomic Condition*, 199.

## References

[R1] Alfer Alexa , and Campos Amy Edwards de . A. S. Byatt: Critical Storytelling. Manchester: Manchester University Press, 2013.

[R2] Brown Alistair . “Uniting the Two Cultures of Body and Mind in A.S. Byatt’s A Whistling Woman’.” The Journal of Literature and Science 1 (2007): 55–72. 10.12929/jls.01.1.04

[R4] Byatt A. S . Babel Tower. London: Vintage, 1997.

[R3] Byatt A. S . A Whistling Woman. London: Vintage, 2003.

[R5] Chomsky Noam . Language and Mind. San Diego: Harcourt, Brace, and World, 1972.

[R6] Coetzee J. M . “En Route to the Catastrophe.” The New York Review of Books. 43:10, 17–19, 1996.

[R7] Dawkins Richard . The Selfish Gene. Oxford University Press: Oxford, 1976.

[R8] De Groot Jerome . Consuming History: Historians and Heritage in Contemporary Public Culture. London: Routledge, 2008.

[R9] De Groot Jerome . Remaking History: The Past in Contemporary Historical Fictions. London: Routledge, 2016.

[R10] Dennett Daniel . Darwin’s Dangerous Idea: Evolution and the Meanings of Life. London: Allen Lane, 1995.

[R11] Diski Jenny . Monkey’s Uncle. London: Phoenix, 1994.

[R12] Dupuy Jean-Pierre . The Mechanization of the Mind: On the Origins of Cognitive Science, trans. M. B. DeBevoise. Princeton: Princeton University Press, 2000.

[R14] Fox Keller Evelyn . Refiguring Life: Metaphors of Twentieth Century Biology. New York: Columbia University Press, 1995.

[R13] Fox Keller Evelyn . Making Sense of Life: Explaining Biological Development with Models, Metaphors, and Machines. Cambridge: Harvard University Press, 2002. 10.1080/00033790310001625820 15366125

[R15] Freeman Elizabeth . Time Binds: Queer Temporalities, Queer Histories. Durham; NC: Duke University Press, 2010.

[R16] Gardner Howard . The Mind’s New Science: A History of the Cognitive Revolution. New York: Basic Books, 1987.

[R17] Gill Josie . Biofictions: Race, Genetics, and the Contemporary Novel. London: Bloomsbury, 2020.

[R18] Glendening John . Science and Religion in Neo-Victorian novels: Eye of the Ichthyosaur. New York; London: Routledge, 2013.

[R19] Hanson Clare . Genetics and the Literary Imagination. Oxford: Oxford University Press, 2020.

[R20] Kay L E , and Kay Lily . “Who wrote the book of life? information and the transformation of molecular biology, 1945-55.” Science in Context 8, no. 4 (1995): 609–34. 10.1017/S0269889700002210 11613275

[R21] Kay Lily . Who Wrote the Book of Life? A History of the Genetic Code. Stanford: Stanford University Press, 2000.

[R23] Lewontin R , Rose S , Kamin L , Rose Steven , and Kamin Leon . “Bourgeois ideology and the origins of biological determinism.” Race & Class 24, no. 1 (1982): 1–16. 10.1177/030639688202400101 11632831

[R22] Lewontin Richard . “Foreword.” In The Ontogeny of Information: Developmental Systems and Evolution, edited by Oyama Susan . Cambridge: Cambridge University Press, 1985.

[R24] Nelson Alondra . “Reconciliation Projects: From Kinship to Justice.” In Genetics and the Unsettled Past: The Collision of Race, DNA, and History, edited by Wailoo Keith , Nelson Alondra , and Lee Catherine . New Brunswick; NJ: Rutgers University Press, 2012.

[R25] Oyama Susan . The Ontogeny of Information: Developmental Systems and Evolution. Cambridge: Cambridge University Press, 1985.

[R26] Pinker Steven . The Language Instinct: The New Science of Language and Mind. London: Allen Lane, 1994.

[R27] Reardon Jenny . The Postgenomic Condition: Ethics, Justice, and Knowledge After the Genome. Chicago: The University of Chicago Press, 2017.

[R28] Richardson Sarah S . “Race and IQ in the postgenomic age: the microcephaly case.” BioSocieties 6, no. 4 (2011): 420–46. 10.1057/biosoc.2011.20

[R29] Rose Nikolas , and Abi-Rached Joelle . Neuro: The New Brain Sciences and the Management of the Mind. Princeton; Oxford: Princeton University Press, 2013.

[R30] Sarkar Sahotra . Genetics and Reductionism. Cambridge: Cambridge University Press, 1998.

[R31] Smith Zadie . White Teeth. London: Penguin, 2002.

[R32] Stevens Hallam , and Richardson Sarah . “Beyond the Genome.” In Postgenomics: Perspectives on Biology After the Genome, edited by Stevens Hallam , and Richardson Sarah . Durham; NC: Duke University Press, 2015.

[R33] Sunder Rajan Kaushik . Biocapital: The Constitution of Postgenomic Life. Durham: Duke University Press, 2006.

[R34] Waugh Patricia . “Mind in Modern Fiction: Literary and Philosophical Perspectives after Darwin.” In The Evolution of Literature: Legacies of Darwin in European Cultures, edited by Saul Nicholas , and James SimonJ , 125–40. Amsterdam: Rodopi, 2011.

